# Quantitative analyses of diameter and running pattern of choroidal vessels in central serous chorioretinopathy by en face images

**DOI:** 10.1038/s41598-020-66858-1

**Published:** 2020-06-12

**Authors:** Hideki Shiihara, Shozo Sonoda, Hiroto Terasaki, Naoko Kakiuchi, Takehiro Yamashita, Eisuke Uchino, Fumiko Murao, Hiroki Sano, Yoshinori Mitamura, Taiji Sakamoto

**Affiliations:** 1 Department of Ophthalmology, Kagoshima University Graduate School of Medical and Dental Sciences, Kagoshima, Japan; 2 Department of Ophthalmology, Tokushima University Graduate School of Medicine, Tokushima, Japan

**Keywords:** Retinal diseases, Translational research

## Abstract

This study was to investigate the choroidal vessels in eyes with central serous chorioretinopathy (CSC) quantitatively. We studied 41 CSC eyes and their fellow eyes, and 41 normal eyes of 41 age-adjusted individuals. En-face optical coherence tomography image of the top 25% slab of Haller’s layer was analyze. The mean vessel area, vessel length, and vessel diameter were calculated. The running pattern of the vessels was quantified and used to determine the degree of symmetry, the “symmetry index”. The vessel area of CSC eyes was not significantly different from that of fellow eyes but significantly larger than that of normal eyes. The vessel length of CSC eyes was not significantly different from fellow eyes but significantly shorter than that of normal eyes. The mean vessel diameter was larger in CSC eyes than in the fellow eyes and the normal eye. The symmetry index was not significantly different in CSC eyes from that of their fellow eyes but was smaller than that of normal eyes. The quantitative analysis showed that eyes with CSC had larger choroidal vessels and asymmetrical vessels running in Haller’s layer.

## Introduction

Central serous chorioretinopathy (CSC) is a disease characterized by serous retinal and pigmented epithelial detachments^1^. Environmental factors such as stress are known to be associated with CSC^2,3^, and recent studies have shown that genetic factors are also associated with CSC^4–6^. Among these, mutations of the *CFH* and *VIPR2* genes have been found to be associated with thicker choroids as well as CSC^7^. Although the choroid is clearly involved in the pathogenesis of CSC^8–12^, the mechanism and role of the choroid in its pathogenesis is still undetermined.

Currently, optical coherence tomography (OCT) is the most used method in researches of the choroid of CSC eyes. However, OCT B-scan images provide only a single slice of the choroid, and it is not always suitable for the analysis of the choroid which is composed mainly of a complex of blood vessels. Recent advances in OCT technology have made it possible to create en face images of multiple layers of the choroid^13^. The strength of en face images is that they represent planar images over a wide area of the choroid. However, there is also a drawback of this method; the image is substantially changed even by a slight change in the plane examined. This drawback is important when examining choroid. Therefore, we have developed a new algorithm to select the same slab of interest of the en face choroidal images using artificial intelligence^14^.

It has been reported that the running pattern of the vessels of Haller’s layer is different between CSC eyes and normal eyes^15–17^. However, these evaluations were done in a subjective manner, and objective and quantitative evaluation methods have not been done. These are critical problems for analyzing the alterations of the choroidal pattern in eyes with CSC. To solve this problem, we have developed an algorithm for the automatic quantification of the running pattern of the choroidal vessels in en face images^18^.

The purpose of this study was to determine whether the characteristics of the choroidal vessels of eyes with CSC differed significantly from that of the fellow eyes and that of age-matched control eyes using an automatic analyzing system. To accomplish this, we compared the area, length, diameter, and running pattern of the vessels in a specific slice of Haller’ layer in eyes with CSC to that of the fellow eyes and age-matched controls. We also examined whether the running pattern of the choroidal vessels may be related to a susceptibility to the development of CSC.

## Results

### Demographic data

**Figure 1:**
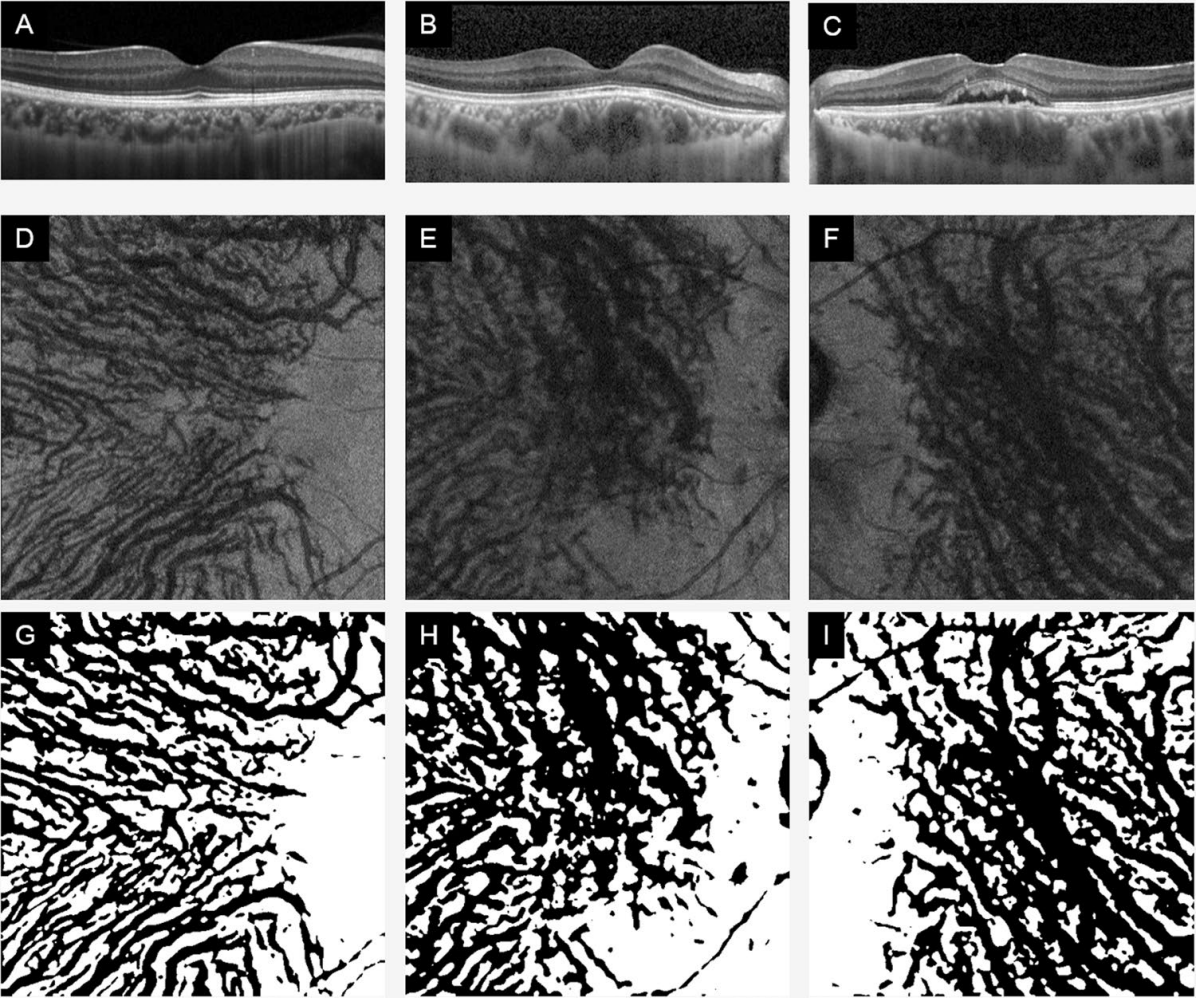
Optical coherence tomographic (OCT) B-scan and en face images of a normal eye and an eye with central serous chorioretinopathy (CSC) and its fellow eye. Representative optical coherence tomographic (OCT) images of normal controls eyes (**A**,**D**,**G**), CSC eyes (**B**,**E**,**H**), and fellow eyes of CSC patient (**C**,**F**,**I**). B-scan images (**A**–**C**), en face images (**D**–**F**), and en face images examined by our software (**G** – **I**).

**Figure 2:**
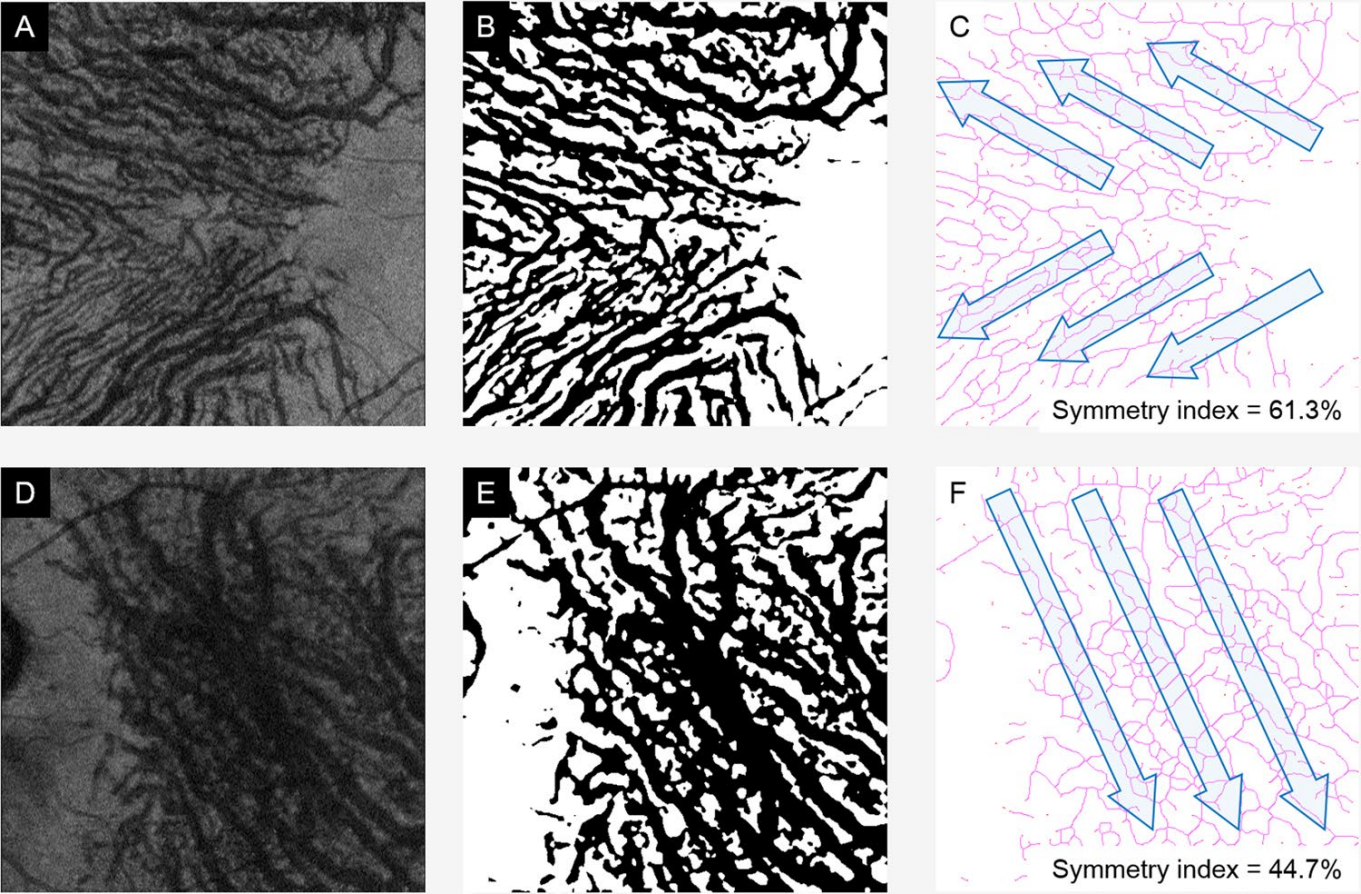
Determination of running pattern of choroidal vessels. The 25% top slab of Haller’s layer was automatically selected (**A**,**D**). It was de-noised (**B**,**E**), and then the symmetry index was calculated (**C**,**F**). (Top row, normal eye and bottom row, CSC eye).

We analyzed 41 eyes with CSC, their fellow eyes and age-matched 41 normal control eyes (Table 1). There were no significant differences in age or sex distribution between the CSC cases and normal subjects. In addition, there were no significant differences in the refractive errors (spherical equivalent) and axial lengths between the CSC eyes and normal eyes (*P* = 0.378, *P* = 0.871), but a significant difference was found in the central choroidal thickness (CCT) (*P* < 0.01). There was no significant difference in equivalent spherical power or axial length between CSC fellow eyes and normal eyes (P = 0.642, P = 0.630), but a significant difference was found in CCT (P < 0.001). There were no significant differences in equivalent spherical power and axial length between CSC eyes and their fellow eyes (P = 0.378, P = 0.871), but significant differences were found in CCT (P = 0.027). Representative OCT images of each group are shown in Figs. 1 and 2. Among the 41 eyes with CSC, 16 had acute CSC and 25 had chronic CSC. No significant differences were found for each choroidal parameter between the two groups. (see Supplementary Table S1).

**Table 1:** Demographic data.

	Control	Fellow	CSC	P value
Age (years)	52.8 ± 11.9	52.0 ± 11.6	52.0 ± 11.6	0.656
Sex (M/F)	21/20	29/12	29/12	0.113
Refractive error (D)	−1.43 ± 1.82	−1.11 ± 1.79	−0.85 ± 2.13	*
Axial length (mm)	24.06 ± 1.14	24.1 ± 1.33	23.93 ± 1.26	*
Central choroidal thickness (μm)	259 ± 87	343 ± 100	391 ± 101	†

*No significant difference between any two of three groups.

^†^Significant difference between any two of three groups (P < 0.05).

### Characteristics of vessels of Haller’s layer

#### CSC eyes vs normal control eyes

**Figure 3:**
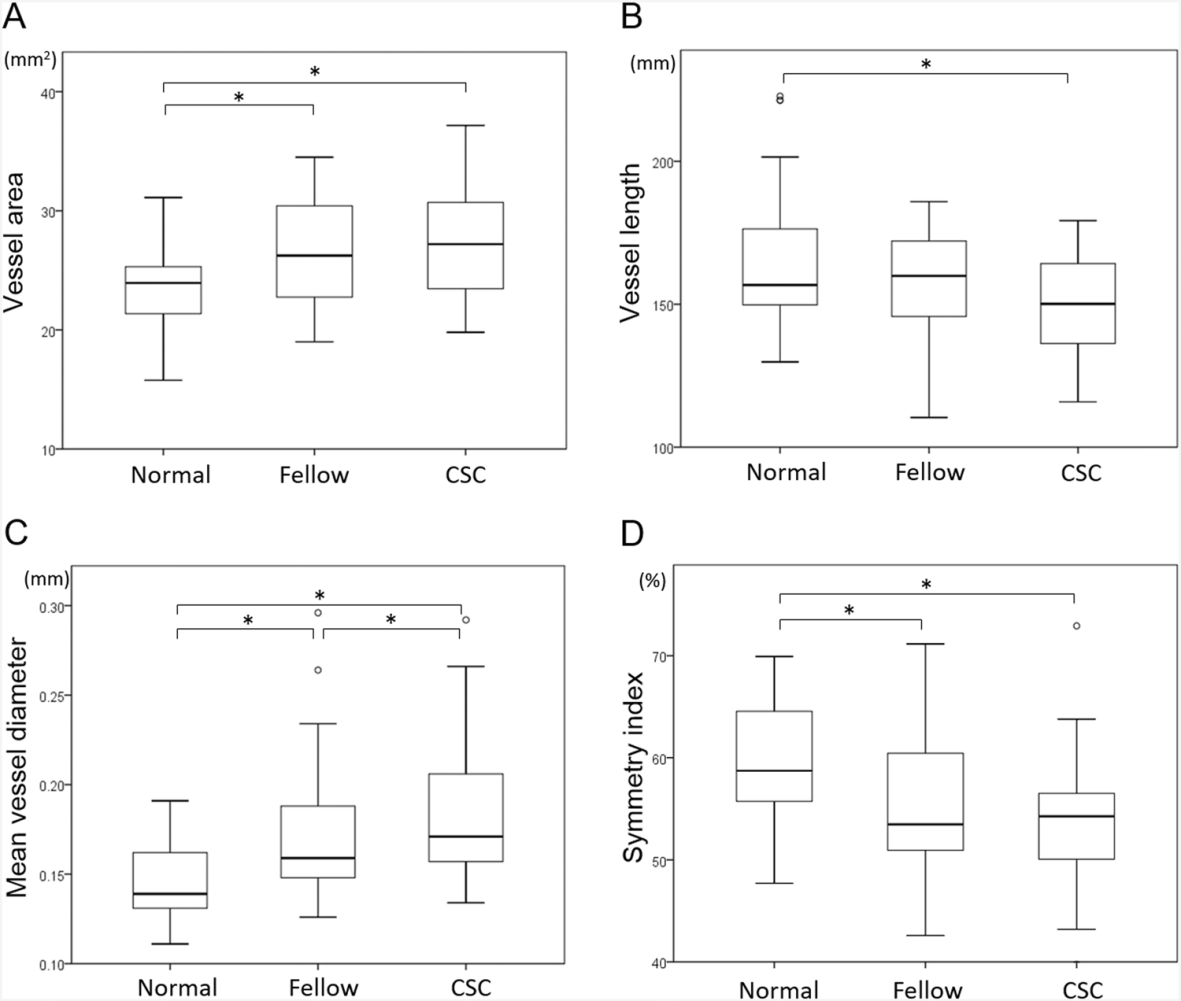
Vessel area (**A**), vessel length (**B**), mean vessel diameter (**C**), and symmetry index (**D**) obtained from en face images of each group. The mean vessel diameter was larger in CSC eyes than fellow eyes (**C**), and the symmetry index is not significantly different between CSC eyes and fellow eyes (**D**). The symmetry index of CSC eyes and fellow eyes was significantly lower than that of normal eyes. *P < 0.05, Wilcoxon singed-rank test.

The vessel area was significantly larger in eyes with CSC (*P* < 0.001), the vessel length was significantly shorter in eyes with CSC (*P* = 0.011), and the mean vessel diameter was significantly larger in CSC eyes (*P* < 0.001). The symmetry index was significantly smaller in eyes with CSC (*P* < 0.001; Fig. 3, Table 2). These findings indicate that the vessels of Haller’s layer are more dilated in CSC eyes than normal eyes, and the vessel running pattern is more asymmetrical than normal eyes.

**Table 2:** Comparison of each parameter.

	Control	Fellow	CSC	P value		
				Control vs Fellow	Control vs CSC	Fellow vs CSC
Vessel area (mm^2^)	23.4 ± 3.3	26.4 ± 4.8	27.4 ± 4.7	0.008	<0.001	0.314
Vessel length (mm)	164.7 ± 24.5	156.6 ± 20.5	150.0 ± 17.1	0.386	0.011	0.080
Mean vessel diameter (mm)	0.144 ± 0.020	0.171 ± 0.038	0.185 ± 0.039	<0.001	<0.001	0.042
Symmetry index (%)	59.4 ± 5.8	55.3 ± 7.2	53.7 ± 6.0	0.007	<0.001	0.568

#### CSC fellow eyes vs normal control eyes

The vessel area was significantly larger in CSC fellow eyes than that of the normal eyes (*P* = 0.008). The vessel length was not significantly different between CSC fellow eyes and normal eyes (*P* = 0.386). The mean vessel diameter was significantly larger in the CSC eyes than that of the normal eyes (*P* = 0.008). The symmetry index was significantly smaller in CSC eyes than that of the normal eyes (*P* = 0.007; Fig. 3, Table 2) These findings indicate that vessels of Haller’s layer are dilated even in CSC fellow eyes as compared to normal eyes, and the vessel running pattern was more asymmetrical than normal eyes.

#### CSC eyes vs CSC fellow eyes

There were no significant differences in the vessel area and the vessel length between eyes with CSC eyes and their fellow eyes (*P* = 0.314, *P* = 0.080). However, the mean vessel diameter was significantly larger in the CSC eyes (*P* = 0.042). The symmetry index was not significantly different between the two groups (*P* = 0.568; Fig. 3, Table 2). These findings indicate that the vessels of Haller’s layer are dilated even in CSC eyes as compared to their fellow eyes, and the vessel running pattern was not different between the two groups.

### Correlation between CCT and choroidal vessel parameters

**Figure 4:**
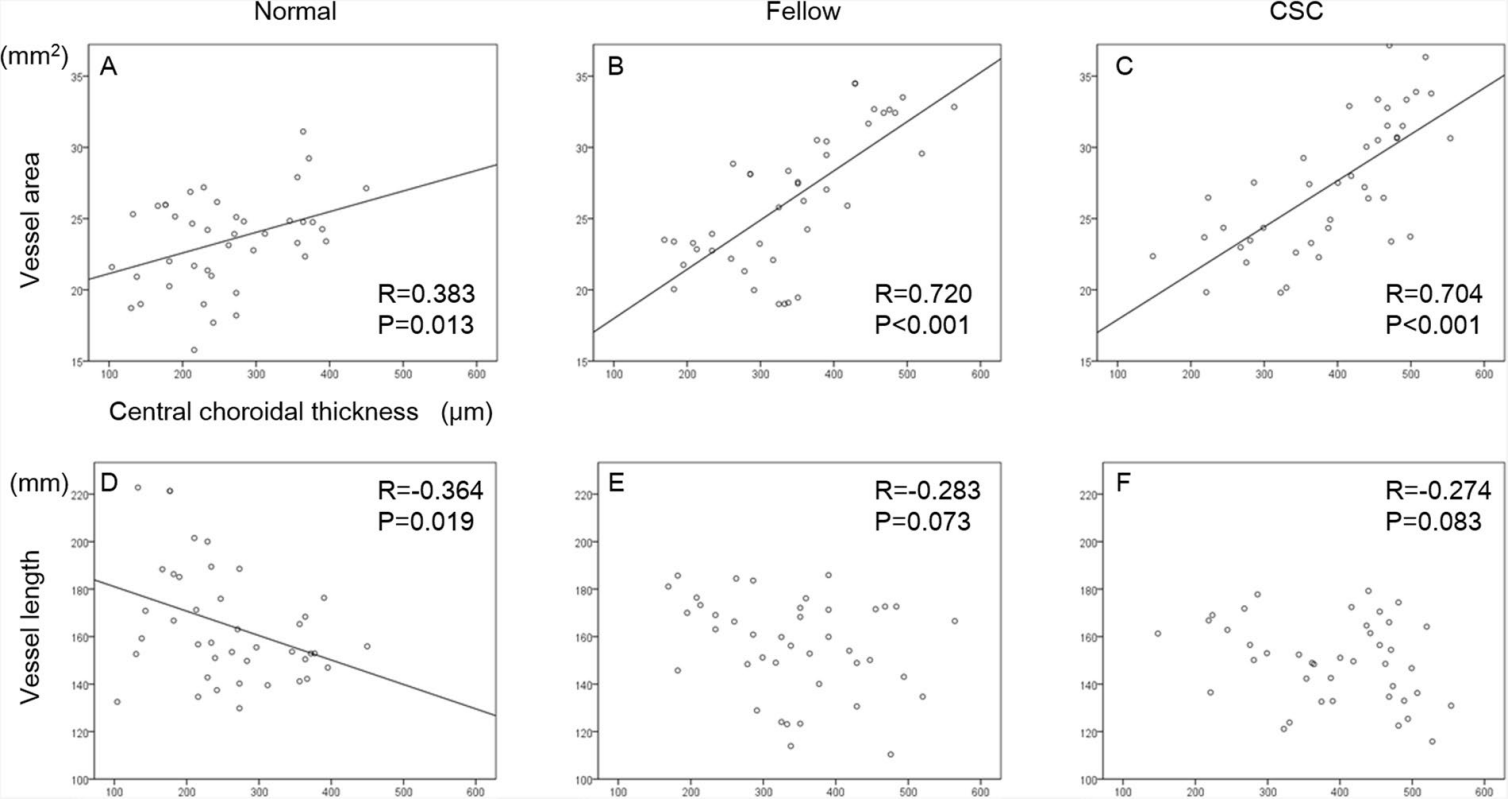
Correlations between vessel area, vessel length, and central choroidal thickness of each group. Scatterplot of normal eyes (**A**,**D**), fellow eyes (**B**,**E**), and CSC eyes (**C**,**F**) are shown. Correlations between the vessel area and central choroidal thickness (CCT) (**A**–**C**), and vessel length and CCT (**D**–**F**) are shown.

**Figure 5:**
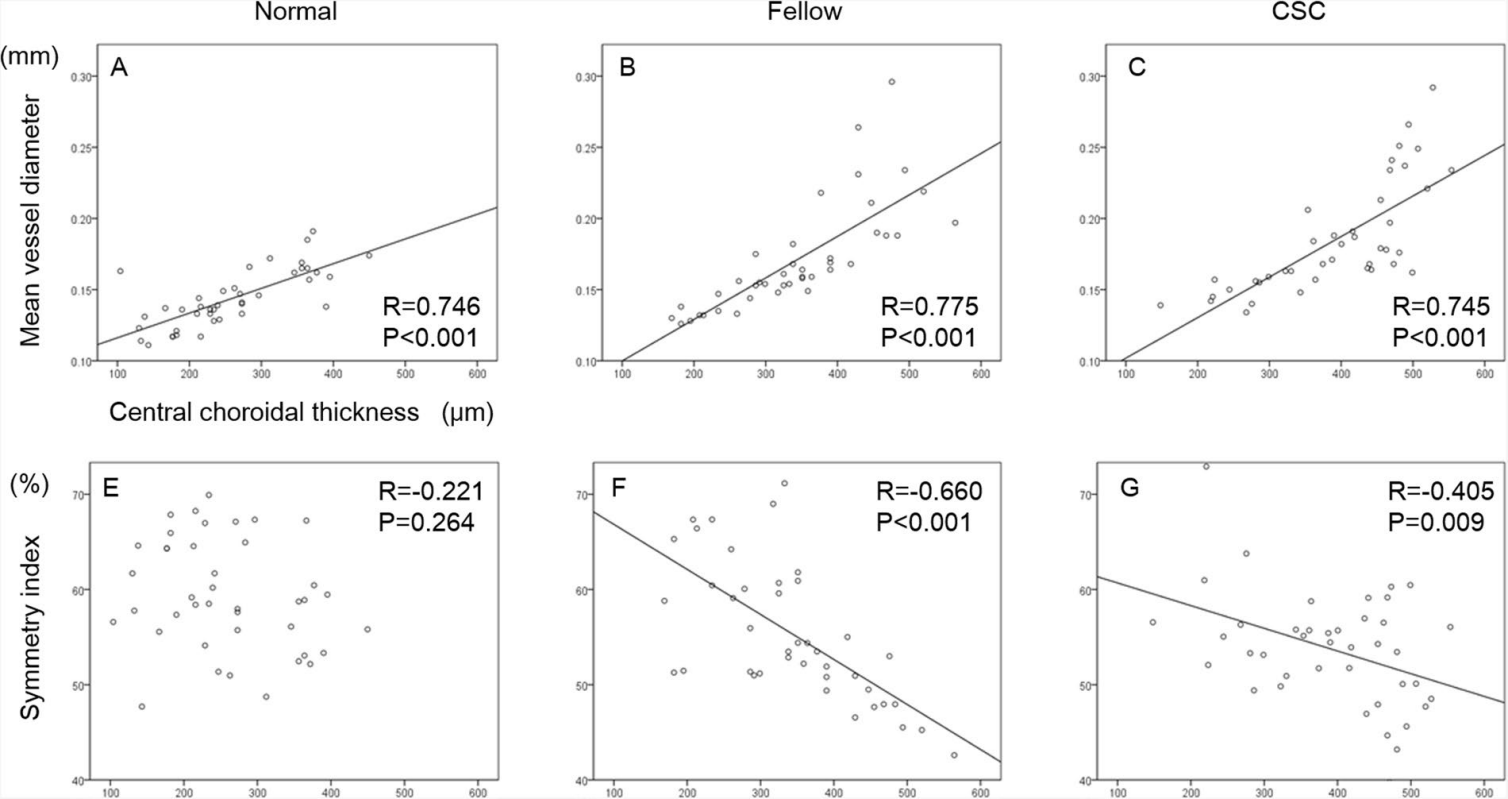
Correlations between the mean vessel diameter, symmetry index, and central choroidal thickness in each group. Scatterplot of normal eyes (**A**,**D**), fellow eyes (**B**,**E**), and CSC eyes (**C**,**F**) are shown. Correlations between the mean vessel diameter and central choroidal thickness (CCT) (**A**–**C**), and symmetry index and CCT (**D**–**F**) are shown.

The correlations between CCT and each parameter from the en face OCT image is shown in Figs. 4 and 5. There was a significant and positive correlation between the vessel area and the CCT in normal eyes, fellow eyes, and CSC eyes (R = 0.383, *P* = 0.019; R = 0.720, *P* < 0.001; R = 0.704, *P* < 0.001, respectively). Although there was a significant negative correlation between the vessel length and CCT in normal eyes (R = −0.364, *P* = 0.019), no significant correlation was found in the fellow eyes and CSC eyes. There was a significant and positive correlation between the mean vessel diameter and CCT in normal eyes, fellow eyes and CSC eyes (R = 0.746, *P* < 0.001; R = 0.775, *P* < 0.001; R = 0.745, *P* < 0.001, respectively). Although there was a significant negative correlation between the symmetry index and CCT in CSC eyes and their fellow eyes (R = −0.660, *P* < 0.001; R = 0.405, *P* < 0.009, respectively), there was no significant correlations in these factors in normal control eyes.

### Cut-off value of mean vessel diameter between CSC eyes and normal eyes

**Figure 6:**
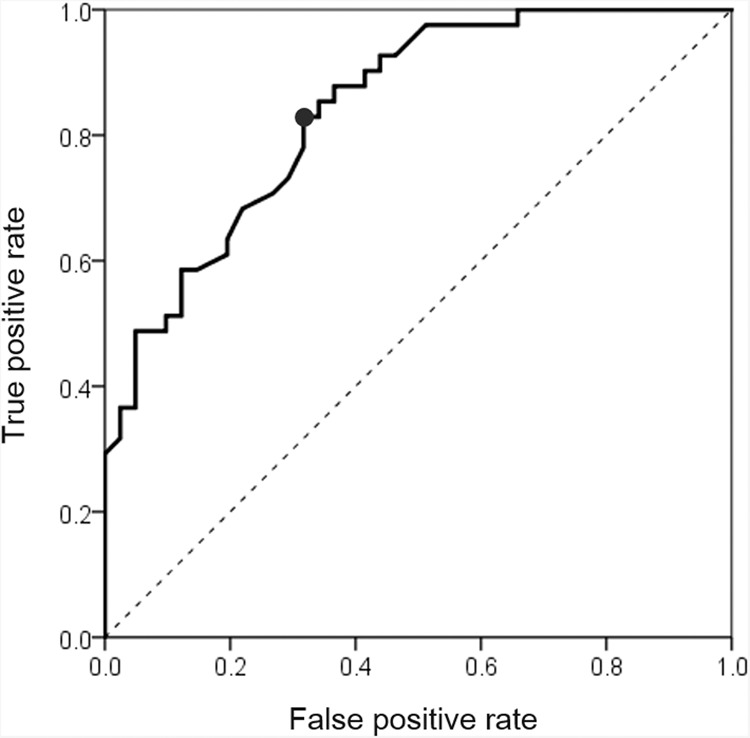
Receiver operating characteristic (ROC) curve and cut off values. The ROC curve is shown for the mean vessel diameter as the independent variable and the presence or absence of the disease as the dependent variable. The highest specificity was obtained when the mean vessel diameter was 0.153 mm.

**Figure 7:**
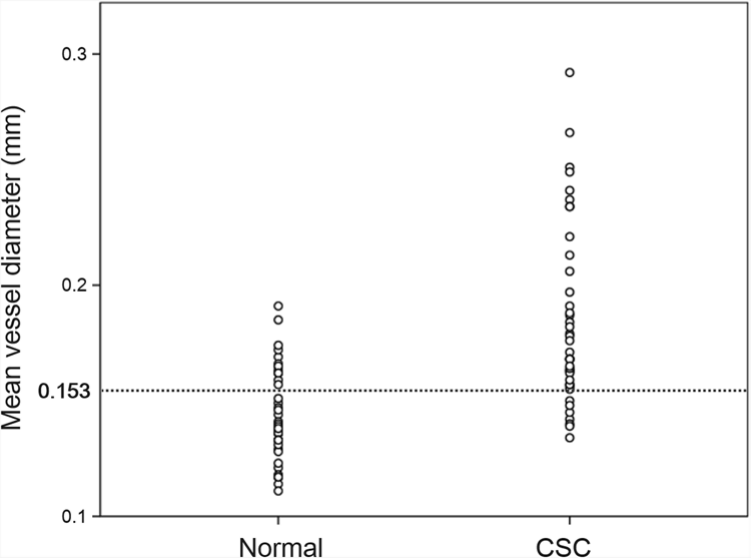
Scatter plot of mean vessel diameter of normal eye and CSC eye. Scatter plots of mean vessel diameter of normal and CSC eyes are shown. The CSC eyes have a larger mean vessel diameter and can be separated by 0.153 mm diameter of cut-off value (see Fig. 6).

The receiver operating characteristic (ROC) curve is shown in Fig. 6 with the mean vessel diameter as the independent variable and the presence or absence of the disease as the dependent variable. The point of the Youden index was at the mean vessel diameter of 0.153 mm. The quadrants where the mean vessel diameter is 0.153 mm and the cut-off value are shown in Table 3. The sensitivity at this point was 82.9% and the specificity was 68.3%. Scatter plots of normal and CSC eyes are shown in Fig. 7.

**Table 3:** A quadrant with mean vessel diameter of 0.153 mm as the cut-off value.

Mean vessel diameter > 0.153 mm	Normal eye (n = 41)	CSC eye (n = 41)
Negative	28 (68.3%)	7 (17.1%)
Positive	13 (31.7%)	34 (82.9%)
Total	41	41

## Discussion

Quantitative evaluations of en face images of Haller’s layer showed that the vessel area was larger in CSC eyes than control eyes, and this was mainly due to enlargements of the vessel diameter rather than a larger number of vessels. The mean vessel diameter in CSC eye was also significantly larger than that of the fellow eyes which is consistent with our earlier study of B-scan images that showed that the lumen area was larger in CSC eyes than that of the fellow eyes^19^. In addition, the mean vessel diameter was significantly larger in the fellow eyes than normal eyes.

It was difficult to determine whether the vessels were enlarged or congenitally large in CSC eye. The results of an earlier study showed that the cause of the larger vessel diameter in CSC can be due to genetic factors^7^. The *CFH* and *VIPR2* genes have been reported to be associated with thickened choroid and the presence of CSC. The *VIPR2* agonist, a vasoactive intestinal peptide, can control the secretion of corticosteroids and has vasodilatory effects in various vascular tissues^20^. These genetic factors might affect the vessel diameter of Haller’s layer congenitally.

**Figure 8:**
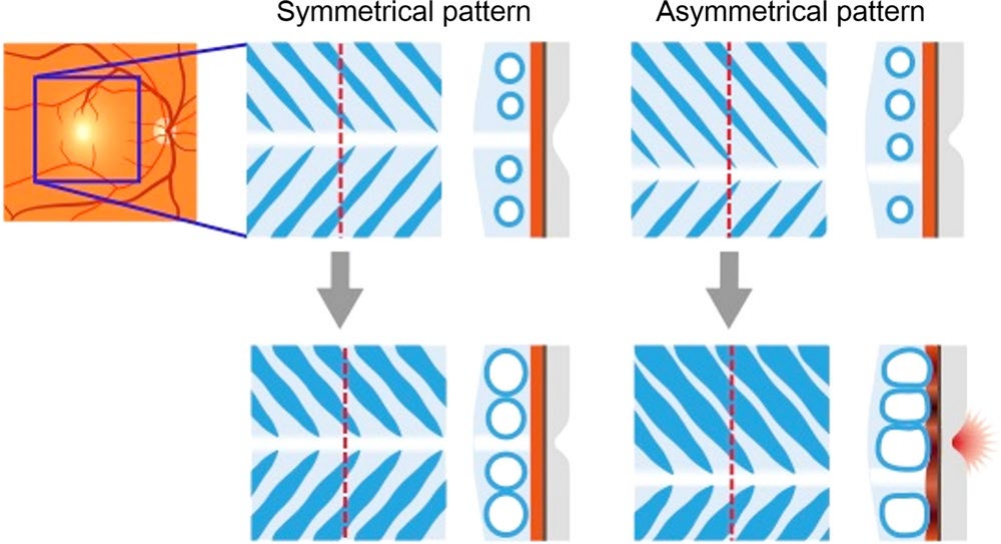
Schematic drawing of hypothetical mechanism of role of vascular running pattern in CSC development. In eyes with vessels of Haller’s layer running symmetrically, the watershed zone exists beneath the subfoveal region. Even when these vessels are dilated, the compression of the choroidal inner layer does not occur. While, in eyes with no symmetry, the watershed zone does not exist beneath the fovea. If the vessels are dilated, dilated choroidal vessels are likely to compress inner choroid, resulting in damage to foveal area. Thus, CSC may be developed.

It was possible to quantify the vessel running pattern with our automatic analysis method (Fig. 2). The results showed that all of the CSC eyes had a lower symmetry index than normal eyes, i.e., an asymmetrical pattern across the horizontal line of the macula. It has been reported that the choroidal vessel running pattern differed significantly between normal eyes and CSC eyes^15–17^. However, the vessel running patterns in these studies were assessed subjectively, and thus the findings were not definitive. Therefore, we developed a new algorithm that determined not only whether the vessel running pattern was symmetrical across the fovea but we also quantified the degree of symmetry by the symmetry index^18^. Our results showed that the symmetry index was not significantly different between the CSC eye and its fellow eye, however it was significantly smaller in the CSC and fellow eyes than that of the normal eyes. Thus, these results suggested that a watershed zone exists in the subfoveal region in CSC and its fellow eye less likely than in normal eyes (Fig. 8).

At present, the CCT is considered to be a major structural parameter of the choroid in the OCT B-scan analysis. By comparing the CCTs with each choroidal parameter from the en face images, a strong positive correlation was observed between the CCT and the vessel area and the mean vessel diameter in all groups. This suggested that the CCT is thicker in cases where vessel diameter is larger and the proportion of larger vessels is higher in Haller’s layer. In other words, it is possible that the CCT is dependent on the thickness of the vessels of Haller’s layer.

On the other hand, a strong negative and significant correlation was found between the CCT and the symmetry index. This indicates that eyes without a watershed area below the fovea have a thicker choroid, and this condition was more common in eyes with CSC. This is consistent with previous qualitative assessments16,17, and it indicates that the predisposing cause of CSC is present not only by the genotype but also by the phenotype of the choroidal vasculature.

Congestion of the choroidal circulation has been suggested to be a factor causing a dilation of the choroidal vessels in CSC eyes^21,22^. In normal eyes, there is a watershed zone just below the macula, and there is little impact of the large vessels on the fovea because large vessels are not present in Haller’s layer near the fovea. However, in eyes that are predisposed to CSC, the vessels of Haller’s layer are present just below the fovea. If a dilatation of the vessels occurs, the inner layer of choroid, such as in the choriocapillaris, may be changed, leading to ischemia and inflammation of the choriocapillaris or the retinal pigment epithelium (RPE) cells. These changes may cause a breakdown of the blood-retinal barrier and exudates may appear. These explanations are consistent with the findings in eyes with CSC^23–26^.

On the other hand, the watershed zone was defined in the earlier studies based on the result of indocyanine green angiography (ICGA), and a close regional relationship between the watershed and CNV was reported^27,28^. However, this method could not distinguish between the area of filling delay and the true watershed zone^23^. Thus, it is necessary to use en-face image to identifying the true watershed zone.

The strength of this research was that the analyses was always performed on the same slab of Haller’s layer that was extracted with good reproducibility by an algorithm using AI^14^. The vessel running pattern and vascular structure in this slab were quantitatively evaluated^18^. This objective method is highly reproducible and repeatable. It is important to note that the mean vessel diameter of the Haller’s layer in eyes with CSC can be distinguished from those of normal eyes using a cut-off value of 0.153 mm. This value may be able to predict an eye likely to develop CSC. Because this finding was obtained non-invasively in a very short time, it can be used clinically with good reliability.

One limitation of this study was that the number of patients analyzed was small and Japanese. Because the prevalence of pachychoroid is not known in all ethnic groups, it is not appropriate to extend the results to other ethnic groups. One may argue that volumetric analysis can be performed more accurately than the analysis of en face images to determine the vascular volume of the choroid^29^. However, volumetric analyses provide objective results of neither the mean vessel diameter nor the vessel running pattern. The techniques used in our study can obtain these important parameters of the choroid.

Currently, the choroidal thickness is the only parameter used as a quantitative factor to evaluate the status of the choroid. However, the present results showed that the diameter and running pattern of vessels in Haller’s layer can also be analyzed quantitatively and objectively. The ability to obtain quantitative and objective values will greatly enhance the validity of the findings in all types of research.

In conclusion, we have developed a method to evaluate the en face images of the vessels in Haller’s layer quantitatively and objectively. This method showed that eyes with CSC have larger and longer diameter vessels. In addition, the running pattern of the choroidal vessels is less symmetrical in eyes with CSC. Because this method is highly reproducible and quantitative, this technique will be useful for understanding not only CSC but also the mechanism for the development of CSC and other retinochoroidal diseases.

## Methods

Institutional Review Board (IRB)/Ethics Committee approval was obtained. This study was approved by the Ethics Committee of Kagoshima University Hospital (Kagoshima, Japan), and was registered with the University Hospital Medical Network (UMIN)-clinical trials registry (CTR). The registration title is “UMIN000031747, Research on retinal/choroidal structure analysis by novel image analysis technique and machine learning.” on March 2018. A detailed protocol is available at, https://upload.umin.ac.jp/cgi-open-bin/ctr/ctr_view.cgi?recptno=R000036250. A written informed consent was obtained from all the subjects after an explanation of the procedures to be used and possible complications. All of the investigative procedures conformed to the tenets of the Declaration of Helsinki.

The subjects included patients with CSC who were examined and treated at either the Kagoshima University Hospital or the Tokushima University Hospital from April 2017 to February 2018. The fellow eyes with no signs of clinical CSC and age-matched healthy eyes were analyzed in the same way and served as controls. The control eyes were those of healthy volunteers with no known ocular diseases who agreed to participate in this study.

The diagnosis of CSC is made by the detection of neurosensory retinal detachments at the macula, one or more leakage spots from the RPE at the acute stage, and a late expansion of the leakages with typical smokestack-shaped fluorescein angiograms. Cases excluded were; bilateral CSC cases, choroidal neovascularization found by ICGA or OCT angiography, and prior photodynamic therapy.

Prior to the measurements, all of the eyes had a comprehensive ocular examination including slit-lamp examinations of the anterior segment of the eye and ophthalmoscopic examinations of the fundus. The intraocular pressure was measured with a pneumo-tonometer (CT-80, Topcon, Tokyo, Japan), and the axial length was measured with the AL-2000 ultrasound instrument (Tomey, Tokyo, Japan). The best-corrected visual acuity (BCVA) was measured after determining the refractive error with an Auto Kerato-Refractometer (RM8900, Topcon).

### Imaging protocol

OCT images were recorded from each eye twice within 1 hour, and the images were processed by two independent examiners who were masked to the clinical findings. Because there are significant diurnal fluctuations of the choroidal morphology, all examinations were done from 13:00 to 16:00 hours on the same day.

The images were obtained as explained in detail^14^. A swept-source OCT device (DRI OCT Triton; Topcon) with a center wavelength of 1050 nm with the 7 × 7 mm 3D scan mode was used. The scanning speed was 100,000 A-scans/sec with 8 μm vertical resolution and 20 μm horizontal resolution. B-scan images were created from 256 horizontal direction of 512 A-scans, and 4 scans were averaged. The EnView software (Topcon) was used for flattening the B-scan images relative to Bruch’s membrane, and averaging was performed with images before and after the flattening. These procedures led to the creation of en face images with a thickness of 2.6 μm for each slab. Then the images were used to create as 512 × 512 pixels bit map images.

### Selection of en face images for analysis

En face images were selected by our automated segmentation program^14^. Briefly, after flattening Bruch’s membrane by the En-View software installed in Topcon Triton, a series of choroid-based en-face images was obtained that ranged from the RPE at the lower end to the sclera. Then the software automatically detected the boundaries of the choriocapillaris, Sattler’s layer, and Haller’s layer. Then, an image corresponding to a depth of the top 25% slab of Haller’s layer was selected for the analyses.

### Measurement of vessel area, vessel length, and mean vessel diameter

The quantification of the choroidal vessels in the en face images was done by our software^18^. Briefly, the selected en face image was binarized and de-noised, and the black area was measured as the vessel area. Then, a thinning of vessels was done by the same software. The total length of the thinned line was measured as the vessel length. The mean vessel diameter was calculated by the vessel area divided the vessel length.

### Symmetry index

The symmetry index is the degree of symmetry of the running pattern of the choridal vessels relatively to a horizontal line across the fovea as described in detail^18^. Briefly, the thinned lines were segmented into line segments at branch point of each vessel. This made it possible to analyze them as a set of line segments in which the thinned vessels have endpoints. Next, the angle between a straight line connecting the ends of the line segments and the X axis was calculated to give the angular information. Thus, these directions from the macula to the upper temporal vortex vein, and from the macula to the lower temporal vortex vein in the lower region were defined as “natural oblique vessels”. The ratio of the total length of these natural oblique vessels to the total length of the entire line segment, i.e., the ratio of the natural oblique vessels, was called the “symmetry index”. In the eye with larger symmetry index, the more vessels run symmetrically across the fovea and the watershed zone exists below the fovea^18^.

In addition, the correlations between the CCT and these parameters of the vessels were calculated for the CSC eyes, fellow eyes, and normal control eyes.

### Cut-off value of mean vessel diameter between CSC and normal eye

To determine the cut-off value of the mean vessel diameter of the vessels of Haller’s layer that classifies CSC eyes and normal eyes, the ROC curves were created with the mean vessel diameter as the predictor variable and the presence or absence of disease as the objective variable. The point where the ROC curve and the line for area under curve (AUC) = 0.500 is the most distant (Youden index) is defined as the cut off value^30^.

### Statistical analyses

All statistical analyses were performed with SPSS statistics 19 for Windows (SPSS Inc., IBM,Somers, NY). Comparisons of the age, axial length, CCT, vessel area, vessel length, mean vessel diameter, symmetry index between the two groups was performed by Mann-Whitney U test. Comparison of the sex differences was done by Chi square tests. Correlation between the CCT and the parameters of the choroid was done by Spearman’s correlation coefficient. A *P* value less than 0.05 was taken to be significant.

## Supplementary information

Dataset 1.
