# Supplementary material for: Quantitative analyses of diameter and running pattern of choroidal vessels in central serous chorioretinopathy by en face images
Source: Sci Rep. 2020 Jun 12;10:9591. doi: 10.1038/s41598-020-66858-1 (PMC7293258; doi:10.1038/s41598-020-66858-1)
Supplement: Supplementary file 1 — Dataset 1. [file 41598_2020_66858_MOESM1_ESM.docx]

**Quantitative analyses of diameter and running pattern of choroidal vessels in central serous chorioretinopathy by en face images**

Hideki Shiihara^1^, Shozo Sonoda^1^, Hiroto Terasaki^1^, Naoko Kakiuchi^1^, Takehiro Yamashita^1^, Eisuke Uchino^1^ Fumiko Murao^2^, Hiroki Sano^2^, Yoshinori Mitamura^2^, Taiji Sakamoto^1^

1) Department of Ophthalmology, Kagoshima University Graduate School of Medical and Dental Sciences, Kagoshima, Japan

2) Department of Ophthalmology, Tokushima University Graduate School of Medicine, Tokushima, Japan

**Supplementary Table S1.** Comparisons of acute and chronic CSC eyes.

|  | Acute CSC (n=16) | Chronic CSC (n=25) | *P* value |
| --- | --- | --- | --- |
| Vessel area (mm^2^) | 27.9 ± 4.7 | 27.0 ± 4.7 | 0.66 |
| Vessel length (mm) | 150.1 ± 14.8 | 149.9 ± 18.6 | 1.0 |
| Mean vessel diameter (mm) | 0.189 ± 0.043 | 0.182 ± 0.037 | 0.61 |
| Symmetry index (%) | 54.7 ± 6.1 | 53.1 ± 5.9 | 0.11 |

(Mann-Whitney U-test)
